# HIV Self-testing Among Previously Diagnosed HIV-Positive People in Khayelitsha, South Africa: No Evidence of Harm but may Facilitate Re-engagement in ART Care

**DOI:** 10.1007/s10461-022-03586-9

**Published:** 2022-03-02

**Authors:** Damian Hacking, Tali Cassidy, Tom Ellman, Sarah Jane Steele, Hazel Ann Moore, Elkin Bermudez-Aza, Xoliswa Nxiba, Eleanor Sopili, Laura Trivino Duran

**Affiliations:** 1 Médecins Sans Frontières, Isivivana Building, 8 Mzala street, Khayelitsha, Cape Town, 7784 South Africa; 2 Division of Public Health Medicine, School of Public Health and Family Medicine, University of Cape Town, Cape Town, South Africa; 3 South African Medical Unit, Cape Town, South Africa; 4 Médecins Sans Frontières, International Office, Cape Town, South Africa; 5 Cape Town City Health, Cape Town, South Africa

**Keywords:** HIV, HIV self-testing, Linkage to care, ART initiation, HIV-positive

## Abstract

In South Africa, where an estimated 34% of nearly 7-million HIV-positive people were not on antiretroviral therapy (ART) in 2019, innovative strategies to diagnose and link people to care are needed. HIV self-testing (HIVST) is one such strategy. However, there is concern that access to HIVST might result in re-testing among people on ART, with a risk of false negative results and disengagement from care. Between November 2017 and December 2018, HIVST kits were distributed at a private pharmacy and at HIV testing outreach events. Each participant was instructed to report their result via SMS and those who did not were followed-up telephonically 10 days later. Electronic medical records of participants were searched for evidence of HIV services 6 months before and after enrollment. Of 1482 participants, 163 (11%) were previously diagnosed HIV-positive prior to taking the test. Of these, 123 reported a result, however 87% reported a negative result. Of the 163 previously diagnosed, 84 were not in ART care prior to the test, with 15 (18%) linking to care post-test. Of 79 who were in ART care prior to the test, 76 (96%) remained in care, even though 51 (67%) had reported a negative result. Overall, 29% of participants reported their result via SMS, and 48% when telephoned. Despite efforts to dissuade them, some previously diagnosed HIV-positive utilised HIVST. For those disengaged from care this may facilitate re-engagement. Self-testing among those already in care, regardless of the reported result, did not disrupt their treatment, and their reasons for doing the test remain unclear.

## Introduction

The WHO recommends innovative strategies to ensure that at least 90% of HIV-positive people know their status (part of the 90-90-90 goals: 90% of the HIV population know their status, of which 90% access treatment, of which 90% suppress their viral load) [1]. This includes the distribution of HIV self-testing kits (HIVST) to close the HIV testing gap in under-reached populations. HIVST kits are either oral-fluid or blood based, and like their traditional counterparts, require confirmatory testing. As such, they have been used in a variety of populations and settings as triage tools to drive potentially positive patients to care [1].

There is concern that HIVST distribution among people on ART might lead to erroneous negative results [2] and subsequent disengagement from care. However, this is likely a matter of providing adequate, accessible instructions, as lay counselors can interpret the results of oral quick self-tests highly accurately [3]. Furthermore, in a study of unassisted HIVST in the South African population, over 95% of participants reported the tests to be easy to use, easy to understand, and felt confident using the test unassisted, 91% correctly interpreted negative results, and 96% correctly interpreted positive results [4]. In another small study in a rural South African setting, untrained lay participants interpreted oral self-tests with 99% sensitivity and 100% specificity (compared to a gold standard of trained rapid blood test users) [5].

In 2016, Medecins Sans Frontieres (MSF) began piloting the distribution of the OraQuick HIV Self-Test (OraSure Technologies Inc) kit in Khayelitsha, a low-income area, home to approximately 500,000 people, in Cape Town [6, 7]. Khayelitsha has a high HIV prevalence and high rates of HIV testing coverage, nevertheless it has yet to achieve the 90-90-90 goals as of 2020 [8]. Due to concerns around false negatives, a feasibility and acceptability study was first performed, which revealed a high confidence in HIVST in the community, as well as areas for strengthening in terms of use and acceptability [9]. These results informed the study design. A conservative distribution approach was taken: an MSF counselor recruited (1) interested participants in the waiting room of a community health centre, and (2) participants who declined fingerprick testing at a fixed community testing site. At both sites participants were asked to confirm that they were not HIV-positive. The use of the test was demonstrated at the recruitment sites, but the tests themselves were conducted by the individual outside of the recruitment site, supplemented with additional pictorial and written instructions. The test proved to be acceptable by the sample population, 27 (4.2%) of which tested positive [7]. However, subsequent analysis revealed that 12 (44%) of those reporting a positive result, and 5% of those reporting a negative result, had a previously recorded positive status in their electronic medical records [7].

Following this study, MSF changed the model of distribution to offering the self-testing kit at all its outreach activities, and at a private pharmacy site within Khayelitsha. The objective was to determine if out-of-facility models of distribution would reach a higher percentage of patients who do not typically test at health facilities (e.g. males and youth). It also sought to describe the retesting rate, and health seeking patterns, of previously diagnosed HIV-positive people who retest using HIVST kits. This short report details the retesting rate of previously diagnosed HIV-positive people, and their prior and subsequent engagement in ART care.

## Methods

### Ethics Approval

This study was performed in line with the principles of the Declaration of Helsinki. Ethics approval for the study was obtained from the University of Cape Town Human Research Ethics Committee (HREC 567/2014) and the MSF Ethics Review Board (Protocol 1432), and permission to conduct the study in the two health facilities was granted by the Western Cape Department of Health.

### Recruitment

Two models of distribution were utilized in this study: distribution in the waiting area of a private pharmacy, and distribution in the vicinity of MSF-led community outreach HIV testing events. Recruitment ran from the 30th of November 2017 to the 18th of December 2018, and in both cases a study recruiter facilitated distribution of the kits. The study recruiter approached individuals and asked them if they would be interested in an HIVST kit. To be eligible the participant had to report being over the age of 18, not on ART, and have a mobile phone number. Interested participants signed an informed consent form, and had their mobile phone numbers and basic demographic data captured onto Telerivet (www.telerivet.com), a web-based platform that allowed for the automation of SMS reminders and free results reporting. The recruiter also demonstrated how to use the testing kit, stressed the importance of not using the kit if already positive, especially if on ART, and provided the participant with written and pictorial instructions in either English or Xhosa, on how to use the kit, and how to voluntarily report their result via the SMS system (free of charge for the user), as well as answered any queries the participant had. These instructions also stressed that previously diagnosed HIV-positive people, especially those on ART, should not use the HIVST kit.

### Tracing and Result Reporting

The Telerivet platform was set up so that participants immediately received an SMS to confirm their successful enrollment. Thereafter, they received a reminder SMS two days later if they had not reported their results, followed by a daily reminder SMS for 7 days. If participants had not reported their result after 10 days, they would be sent a final SMS request, followed by telephonic tracing, up to three times, in order to determine their test result. At the end of the study, all participants who had not reported a result were telephonically traced in one final attempt to obtain their results.

### Clinical Data Sources

In line with the study protocol, and in order to monitor patient outcomes, all participants were manually searched for on the online portal of the National Health Laboratory System (NHLS). Dates of viral load and CD4 count tests were recorded for all participants. At the end of the study period, participant names and dates of birth were securely provided to the Western Cape’s Provincial Health Data Center (PHDC), in order to obtain clinical data of participants. The PHDC links patient data from multiple sources, including laboratory data, clinic data and pharmacy dispensing [10]. The researchers were provided with a dataset with the minimum data required (dates of ART dispensing, viral loads, and CD4 tests of linked participants), linked to a unique study identifier for each participant.

### Measures

Participants were considered to have reported a result if they sent an unambiguous text message or verbally reported their result when phoned. Participants were considered to be previously diagnosed with HIV if there was any evidence of them having received ART, or having CD4 counts or viral load laboratory tests, before the date that they were enrolled in the study. Participants were considered to be in ART care at the time of HIVST if they had received ART or had a viral load result in the 6 months prior to study enrolment. Participants were considered linked to, or retained in, ART care if they received ART or had CD4 testing within 6 months after study enrolment.

### Analysis

Data from the different data sources were combined and described using Stata 14 [11]. We describe baseline participant characteristics stratified by study site, and numbers and proportions linking to ART care stratified by prior HIV and ART status and reported results.

## Results

In total 1482 participants were enrolled, 52% within the community, and 48% at the pharmacy (Table 1). Overall, 425 (29%) participants reported a result via SMS, and 712 (48%) reported their result when telephoned; the remaining 23% of participants did not report a result. Due to implementation constraints, 543 (37%) participants experienced a delay in receiving their reminder SMSes. The longer the delay in receiving the SMS reminders, the less likely the participant was to self report their result (data not shown), and of the 94 who did not receive any SMS reminders, 93 (99%) did not SMS their results.

**Table 1:** Participants baseline characteristics and reported results by recruitment site

	Community distribution	Pharmacy-based distribution	Total
N	774	708	1482
Male (%)	240 (31)	184 (26)	430 (29)
Under 25 years old (%)	334 (43)	212 (30)	534 (36)
Median age (IQR)	26.4 (22–32)	28.7 (24–35)	27.4 (23–33)
Reported any result (%)	558 (72)	580 (82)	1141 (77)
Reported by SMS (%)	255 (33)	170 (24)	425 (29)
Median days to reply (IQR)	2 (0–7)	2 (1–5)	2 (0–6)
Reported positive (%)	24 (3)	9 (1)	33 (2)

Table 2 subdivides results reporting and linkage to ART care according to the participants’ prior knowledge of HIV and ART care status. Overall, 11% (163/1482) of those enrolled in the study had a record of prior HIV diagnosis. These previously diagnosed positives made up 36% (12/33) of all positive results reported. Of the previously diagnosed positives, 34% (55/163) had no prior evidence of being on ART, 18% (29/163) had prior ART evidence but not in the 6 months before enrolment, while 48% (79/163) had evidence of ART care within 6 months before enrolment. Of these, 96%, (76/79) had evidence of continued ART care within the 6 months after enrolment.

**Table 2:** Linkage to HIV care by prior HIV and ART status and reported results

Reported result	N	n linked	% linked
*No evidence of ART care or previous positive diagnosis before test*			
Unreported	288	4	1
Reported negative	1000	10	1
Reported positive	21	2	10
Unclear	10	0	0
Total	1319	16	1
*Previously diagnosed positives, previous ART*^a^*, not in ART care 6 months prior*			
Unreported	8	1	13
Reported negative	19	4	21
Reported positive	2	1	50
Unclear	0	0	0
Total	29	6	21
*Previously diagnosed positives*^b^*, no evidence of prior ART*			
Unreported	13	4	31
Reported negative	35	5	14
Reported positive	5	0	0
Unclear	2	0	0
Total	55	9	16
*Previously diagnosed positives, in ART care* < *6 months prior*			
Unreported	19	18	95
Reported negative	53	51	96
Reported positive	5	5	100
Unclear	2	2	100
Total	79	76	96

^a^Evidenced by previous viral load result or ART dispensed

^b^Evidenced by previous positive test or CD4 count result

The majority, 66%, (107/163) of previously diagnosed positives, reported a negative HIVST result. Furthermore, the proportion of HIV-positive participants reporting a negative result was consistent across those diagnosed but not on ART (64%, 35/55), currently disengaged from ART care (66%, 19/29), and currently engaged in ART care groups (67%, 53/79).

Amongst those with no prior evidence of HIV-positive status, 2% (21/1319) reported a positive result. Additionally, of the 1000 participants who reported a negative result, 10 (1%) subsequently linked to care and were confirmed to be HIV-positive.

While linkage rates among new positives appeared low at 10% (2/21), 18% (15/84) of those previously diagnosed but not on ART were found to relink to care; 16% (9/55) among those never known to have started ART and 21% (6/29) among those with evidence of prior ART but disengaged at the time of HIVST.

## Discussion

The Western Cape province of South Africa, with a comprehensive electronic data system in the public sector and a high HIV prevalence, provided a unique opportunity to identify prior HIV and ART status of HIVST kit-users and the linkage after testing. We identified three groups of participants that warrant further investigation: (1) participants with prior evidence of HIV-positive status (n = 163); (2) participants with prior evidence of HIV-positive status who reported negative HIVST results (n = 107); (3) participants reporting a negative result and subsequently linked to care and confirmed to be HIV-positive (n = 10).

The first group, participants with prior HIV-positive status, constituted a large proportion (11%) of the study population, despite the fact that during recruitment and the informed consent process participants were instructed not to take the test if they were known-positive, informed of the potential for false negatives, and a sticker was affixed to the testing kit instructing those on ART not to use the test. It is possible that previously diagnosed HIV-positive participants may have simply been curious to try out the HIVST, or perceived participation as beneficial (e.g. ‘reassurance’ that their previous test result was correct) [12]. This confirmation of positive status might also have been a gateway to re-engaging in services: 18% (15/84) of those previously diagnosed but not on ART linked to care. This is in line with other findings that users who had tested for HIV previously were more likely to link to care after HIVST, compared to users who were testing for the first time [13]. The particularly high proportion of known positives overall may also reflect the high coverage of HIV services in a high HIV prevalence setting (31% antenatal prevalence in 2017) [14], and may be lower in other contexts [13].

The second group, previously diagnosed positives reporting a negative HIVST result, raises concerns about false-negative results and disengagement. We cannot rule out this possibility, however, as previously stated, literacy around HIVST and results interpretation is high in this population. Participants may have been avoiding further follow-up by reporting a negative result, or may have taken the test for a friend or partner. There were no notable differences in linkage between those reporting negative, positive, or not reporting, suggesting that negative results reporting was due to misreporting rather than false-negative results due to being on ART. Reassuringly, of the 53 on ART at recruitment who reported a negative result, 96% were still in care 6 months later.

The third group constitutes ten patients who reported a negative result but linked to HIV care. We cannot ascertain linkage rates for this group, as we do not know how many tested positive, reported negative, and did not link. However, if the proportion linked was similar to those reporting positive (9.5%), then approximately 105 of the 1000 participants reporting a negative result could in fact be newly diagnosed positives. Furthermore, if we had only followed up those reporting a positive result to determine linkage to care, then only 2 out of the 16 newly diagnosed participants who linked to care would have been attributed to HIVST, vastly underestimating its impact. It is unlikely that participants used the test kit incorrectly or misinterpreted their test results, as high literacy on the use of HIVST is well documented [3–5], and additional local-language pictorial instructions on using the kit and interpreting the results were provided. It therefore remains unclear why these newly diagnosed positives, including the ones who subsequently linked to care, reported a negative result.

Our findings were comparable to the facility-based study previously reported [7]. The distribution outside of the facility reached a higher proportion of males (29 vs 5.2% in the facility-based study), but had similar youth reach (36 vs 24%). Across all sites a total of 2121 were enrolled, of whom 10% (202/2121) were previously diagnosed HIV-positive (6.1% (39/639) in the facility study, 11% (163/1482) in the outside of facility study). Of the previously diagnosed positives who reported a result, 64% (129/202) reported a negative result—57% (22/39) in facility compared to 66% (107/163) outside of facility. Facility clients may have been more hesitant to falsely report a negative result to a counsellor at a facility where they regularly sought care [15].

Despite electronic linkage, under-ascertainment of previously diagnosed HIV-positives and linkage is likely if participants tested for HIV or linked to ART care in another province, using a different name or date of birth, or used the private sector. In addition, it was not possible to confirm that the participant who enrolled in the study was the same person who used the HIVST, as participants may have given the test to partners, family, or friends. This may be especially true for the known positives. Participants may also have elected to take an HIVST due to a very recent potential exposure, and may have tested prior to seroconversion, which would explain those reporting a negative result yet subsequently linking to ART care within 6 months. Lastly, the majority of results in this cohort were obtained by phone call only after the participant had failed to report a result via SMS, and this, combined with the SMS reminders every second day, may have incentivized the participants to report a result when they did not want to and in so doing contributed to the reporting of false results—recall that 99% of those who did not receive any SMSes did not report a result. While these potential explanations do not describe the researchers’ intended use of the HIVST kits, they suggest participants are nonetheless engaging with and confronting their HIV status (or helping others to do so). Further research, such as qualitative interviews with participants, is needed to understand these unintended (but potentially positive) uses of HIVST kits.

## Conclusions

This study has demonstrated that the majority of HIVST users reporting a positive result were aware of their status and on treatment at some point prior to the test. The majority of HIV-positive individuals testing remained in care, despite many reporting negative results. A significant number of those who were disengaged from care re-engaged. These findings suggest the need for further research on reasons for testing and re-testing with HIVST, and barriers to disclosure of status before and after testing. Discouragement of re-testing among those who know their status may not be effective, and further research is needed to investigate the potential role of HIVST in supporting re-engagement with care.
